# *N*‐glycan signature of serum immunoglobulins as a diagnostic biomarker of urothelial carcinomas

**DOI:** 10.1002/cam4.3727

**Published:** 2021-01-16

**Authors:** Hirotake Kodama, Tohru Yoneyama, Toshikazu Tanaka, Daisuke Noro, Yuki Tobisawa, Hayato Yamamoto, Shinichiro Suto, Shingo Hatakeyama, Kazuyuki Mori, Takahiro Yoneyama, Yasuhiro Hashimoto, Ikuko Kakizaki, Shigeyuki Nakaji, Chikara Ohyama

**Affiliations:** ^1^ Department of Urology Hirosaki University Graduate School of Medicine Hirosaki Japan; ^2^ Department of Glycotechnology Center for Advanced Medical Research Hirosaki University Graduate School of Medicine Hirosaki Japan; ^3^ Department of Urology Mutsu General Hospital Mutsu Japan; ^4^ Department of Social Medicine Hirosaki University Graduate School of Medicine Hirosaki Japan

**Keywords:** capillary‐electrophoresis, diagnostic biomarker, immunogloburins, *N*‐glycosylation signatures, urothelial carcinoma

## Abstract

Discriminating between urothelial carcinoma (UC), including bladder cancer (BCa) and upper urinary tract UC (UTUC), is often challenging. Thus, the current study evaluated the diagnostic performance of *N*‐glycosylation signatures of immunoglobulins (Igs) for detecting UC, including BCa and UTUC. *N*‐glycosylation signatures of Igs from serum samples of the training cohort, including 104 BCa, 68 UTUC, 10 urinary tract infection, and 5 cystitis cases, as well as 62 healthy volunteers, were measured retrospectively using automated capillary‐electrophoresis‐based *N*‐glycomics. UTUC or BCa scores were then established through discriminant analysis using *N*‐glycan signatures of Igs. Diagnostic performance was evaluated using the area under receiver operating characteristics curve (AUC) and decision curve analyses (DCA). Our result showed that BCa and UTUC scores for discriminating BCa (AUC: 0.977) and UTUC (AUC: 0.867), respectively, provided significantly better clinical performance compared to urine cytology, gross hematuria, or clinical T1 cases. DCA revealed that adding BCa and UTUC scores to gross hematuria status was the best combination for detecting UC and avoiding the need for more intervention without overlooking UC (risk threshold: 13%–93%). The UC nomogram based on the combination of gross hematuria, UTUC score, and BCa score could detect UC with an AUC of 0.891, indicating significantly better performance compared to gross hematuria status in the validation cohort (251 patients). The limitations of this study include its small sample size and retrospective nature. The UC nomogram based on gross hematuria and *N*‐glycosylation signatures of Igs can be a promising approach for the diagnosis of UC.

## Graphical abstract

We evaluated the diagnostic performance of Bladder cancer (BCa) and upper urinary tract urothelial carcinoma (UTUC) discrimination scores that we developed based on N‐glycosylation signatures of immunoglobulins. The BCa score and UTUC score provided significantly better clinical performance than urine cytology negative or gross hematuria negative or clinical T1 cases.

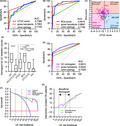

## 1 INTRODUCTION

Urothelial carcinoma (UC) is the eighth most lethal cancer among men in the United States.^1^ The majority of UC cases originate from the bladder, beginning as bladder cancer (BCa), while between 5% and 10% of cases originate from the ureter or renal pelvis [collectively called upper urinary tract UC (UTUC)].^2^, ^3^ The latter have been associated with a worse prognosis than cases originating from BCa. The most common symptom of UC has been visible or nonvisible hematuria (70%–80%),^4^, ^5^ which can be detected through standard diagnostic tools for UC, including urine cytology, urinary tract imaging, and cystoscopy. However, 60% of UTUC cases are already invasive at the time of diagnosis^6^, ^7^ given the unreliability of urine cytology for detecting of early stage UC and UTUC, as well as the difficulty of visualizing small tumors using imaging modalities, such as ultrasound or computed tomography. Thus, a more powerful, less invasive biomarker that allows for the initial diagnosis of UC would be substantially beneficial to the field and may improve patient outcomes.

Glycosylation is a common posttranslational modification that is important for various biological functions. Our previous study demonstrated that high‐throughput, comprehensive, and quantitative glycoblotting‐based *N*‐glycomics combined with mass spectrometry (MS) can be a promising method for screening glycans to identify diagnostic and prognostic markers of several cancers.^8^, ^9^, ^10^, ^11^, ^12^, ^13^, ^14^ Moreover, we reported that the presence of a combination of several serum *N*‐glycans (termed the *N*‐glycan score) can be a novel serum marker for UC, as well as UTUC, and is able to detect 93% of UC cases, making it far more specific than classic urine cytology.^15^ Based on this, our group reported that the detection of aberrant *N*‐glycosylation profiles of immunoglobulins (Igs) through *N*‐glycomics may be useful for diagnosing UC.^16^ However, the aforementioned MS‐based *N*‐glycomics approach requires 48–72 h to determine the *N*‐glycans on Igs, is insufficiently versatile for clinical application, and is a rather expensive.

To overcome the limitations of our approach, we investigated the application of rapid capillary electrophoresis‐light‐emitting‐diode‐induced fluorescence (CE‐LIF)‐based *N*‐glycomics to quantify Ig *N*‐glycan structures in serum samples of healthy volunteers (HV) and patients with BCa, UTUC, urinary tract infections (UTI), and cystitis (CYS). From this, discriminatory scores for BCa and UTUC (BCa and UTUC scores, respectively) were established based on the *N*‐glycan signatures of Igs associated with BCa and UTUC, respectively. Decision curve analysis (DCA) revealed that assessing gross hematuria status, BCa score, and UTUC score could reduce the need for further intervention. As such, we established a UC diagnostic nomogram for clinical use, which could detect UC with higher specificity than gross hematuria or urine cytology. The use of CE‐LIF incorporating an automated *N*‐glycomics platform can more efficiently achieve assay performance specifications required in clinical laboratories compared to existing methodologies.

## 2 MATERIALS AND METHODS

### 2.1 Study design and participant recruitment

**FIGURE 1:**
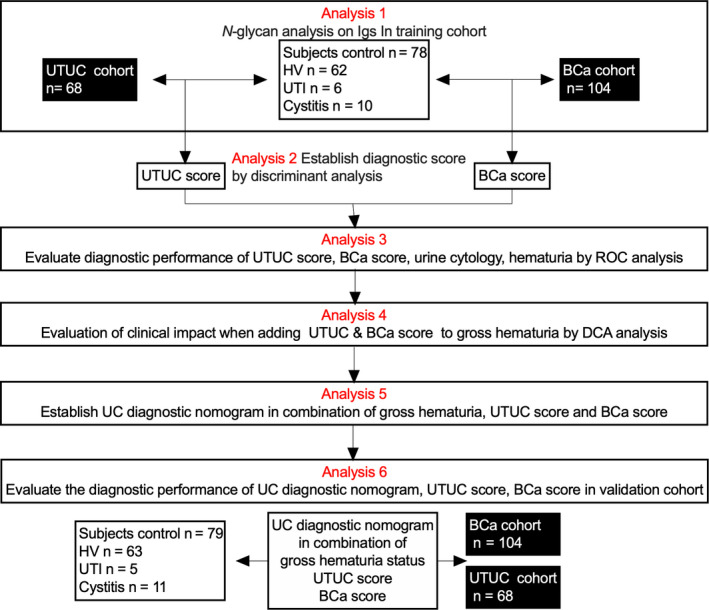
Flow diagram of the study design. Training cohort serum samples collected from patients with UTUC, BCa, CYS, and UTI, as well as from healthy volunteers, were subjected to *N*‐glycan analysis of immunoglobulins (Analysis 1). Discriminant analysis for the detection of UTUC and BCa was conducted using serum levels of the 26 types of *N*‐glycans on Igs analyzed for each patient, after which UTUC and BCa scores were established (Analysis 2). The diagnostic performances of the UTUC score, BCa score, urine cytology, and gross hematuria status were compared using ROC analysis (Analysis 3). The clinical impact of adding the UTUC and BCa scores to the base diagnostic model was evaluated using DCA (Analysis 4). The urothelial carcinoma diagnostic nomogram was established based on a combination of UTUC and BCa scores (Analysis 5). The diagnostic performance of the UTUC and BCa scores and clinical impact of the UC nomogram were evaluated using ROC analysis and DCA in the validation cohort (Analysis 6). BCa, bladder cancer; DCA, decision curve analysis; HV, healthy volunteer; ROC, receiver operating characteristic; UTI, urinary tract infection; UTUC, upper urinary tract urothelial carcinoma; Igs, immunoglobulins; CYS, cystitis.

A flow diagram for this retrospective observational study is presented in Figure 1. Training and validation cohort patients with UC (UTUC or BCa), UTI, or CYS who were treated at Hirosaki University Hospital and Mutsu General Hospital between June 2007 and July 2020 were recruited. Those who received previous clinical treatment were excluded. Serum samples from patients with UTUC, BCa, CYS, and UTI were obtained upon UC diagnosis or initial treatment. Subjects from community‐dwelling populations involved in the Iwaki Health Promotion Project were also recruited as HVs.^15^, ^17^ The present study was conducted in accordance with the ethical standards of the Declaration of Helsinki. The study design and protocols were approved by the Institutional Review Board of Hirosaki University Graduate School of Medicine (“study about carbohydrate structure change in urological disease”; approval number: 2019–099, approval date: 13 March 2020). Written or verbal informed consent was obtained from all serum donors.

### 2.2 Assessments

*N*‐glycan signatures from serum samples of the training cohort were analyzed using CE‐LIF‐based *N*‐glycomics (Analysis 1 in Figure 1). We then evaluated the diagnostic performance of UTUC and BCa scores (Analysis 2 in Figure 1), as well as the correlation between UTUC/BCa scores and several diagnostic factors, including clinical T stage, urine cytology, and gross hematuria (Analysis 3 in Figure 1). The clinical impact of UTUC and BCa scores in combination with gross hematuria status was analyzed using DCA (Analysis 4 in Figure 1). The UC nomogram in combination with gross hematuria, UTUC score, and BCa score was then established using training cohort data (Analysis 5 in Figure 1). The clinical impact of the BCa score, UTUC score, and UC nomogram in the validation cohort was analyzed using receiver operating characteristics curve (ROC) analysis and DCA (Analysis 6 in Figure 1).

### 2.3 Serum samples and patient information

All serum samples were stored at −80°C until use. All tumors were staged according to the 2017 Tumor‐Node‐Metastasis Classification, 8th edition.^18^ Histological classification of UC was carried out according to the World Health Organization 1973 and 2004 grading systems.^19^ Urine cytology classification was performed according to the guidelines of The Paris System working group.^20^ Data regarding age, sex, gross hematuria, urine cytology class, tumor location, tumor grade, and clinical stage were recorded for all subjects.

### 2.4 Purification and quantification of immunoglobulins in sera

**FIGURE 2:**
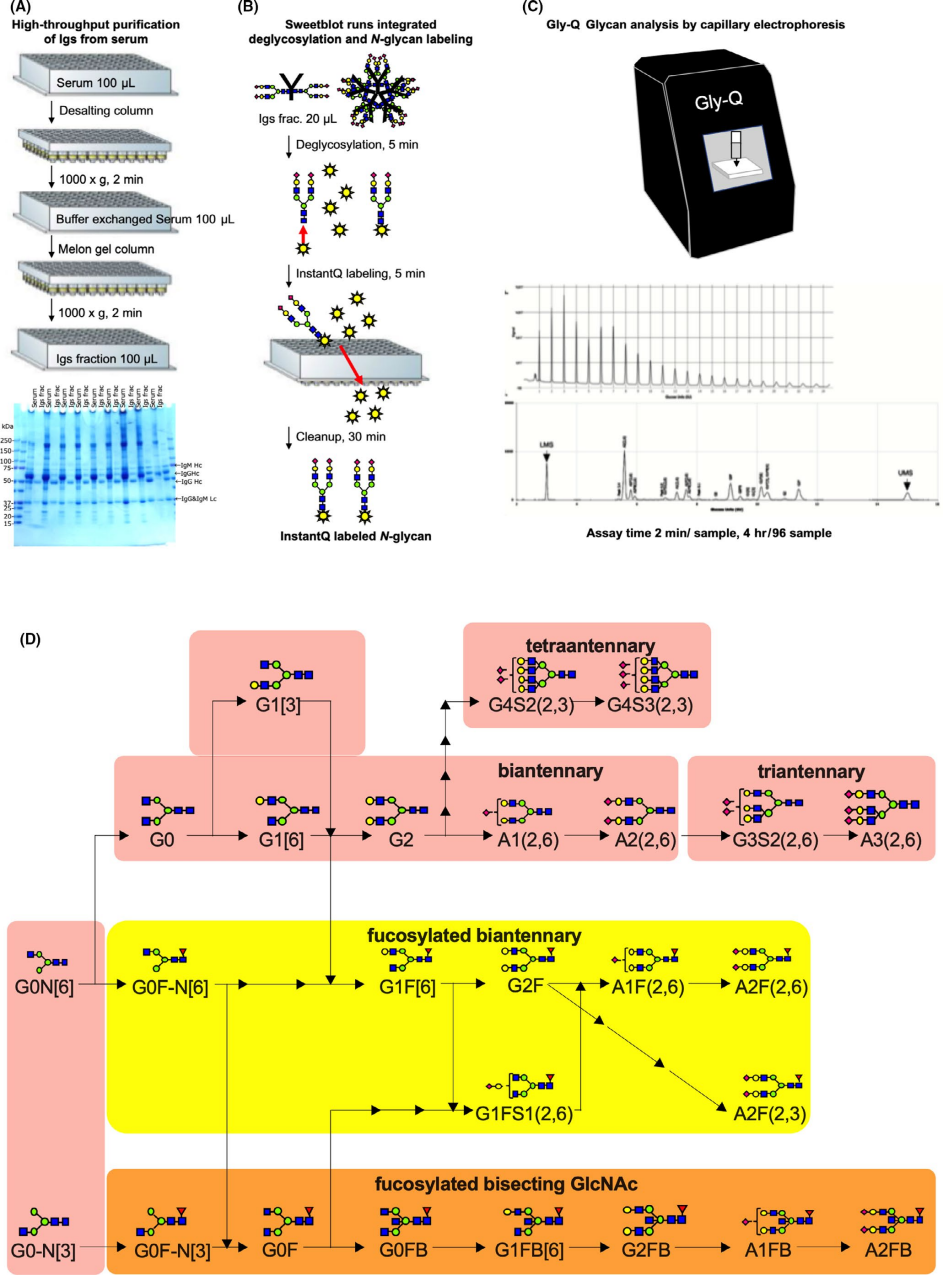
Schematic of the workflow for capillary‐electrophoresis‐based high‐throughput clinical *N*‐glycan analysis. (A) In total, 100‐µL aliquots of serum samples were applied to high‐throughput purification of Igs. The gel image shows representative Coomassie brilliant blue‐stained band patterns of the whole serum and purified Ig samples on sodium dodecyl sulfate‐polyacrylamide gel. (B) In total, 22‐µL aliquots of purified Ig samples were applied to the SweetBlot™ instrument (System Instruments, Hachioji, Japan) for deglycosylation of Igs and InstantQ labeling of the *N*‐glycans. (C) Capillary electrophoresis‐based *N*‐glycan analysis of InstantQ‐labeled *N*‐glycans from purified Ig samples. (D) A total of 26 *N*‐glycans were identified in the purified Ig samples and were indicated in the synthetic pathway of *N*‐glycans. Identified *N*‐glycan structures are indicated by monosaccharide symbols: yellow circles, galactose (Gal); green circles, mannose (Man); blue squares, *N*‐acetylglucosamine (GlcNAc); red triangle, fucose (Fuc) and purple diamonds, sialic acid. Igs, immunoglobulins.

**FIGURE 3:**
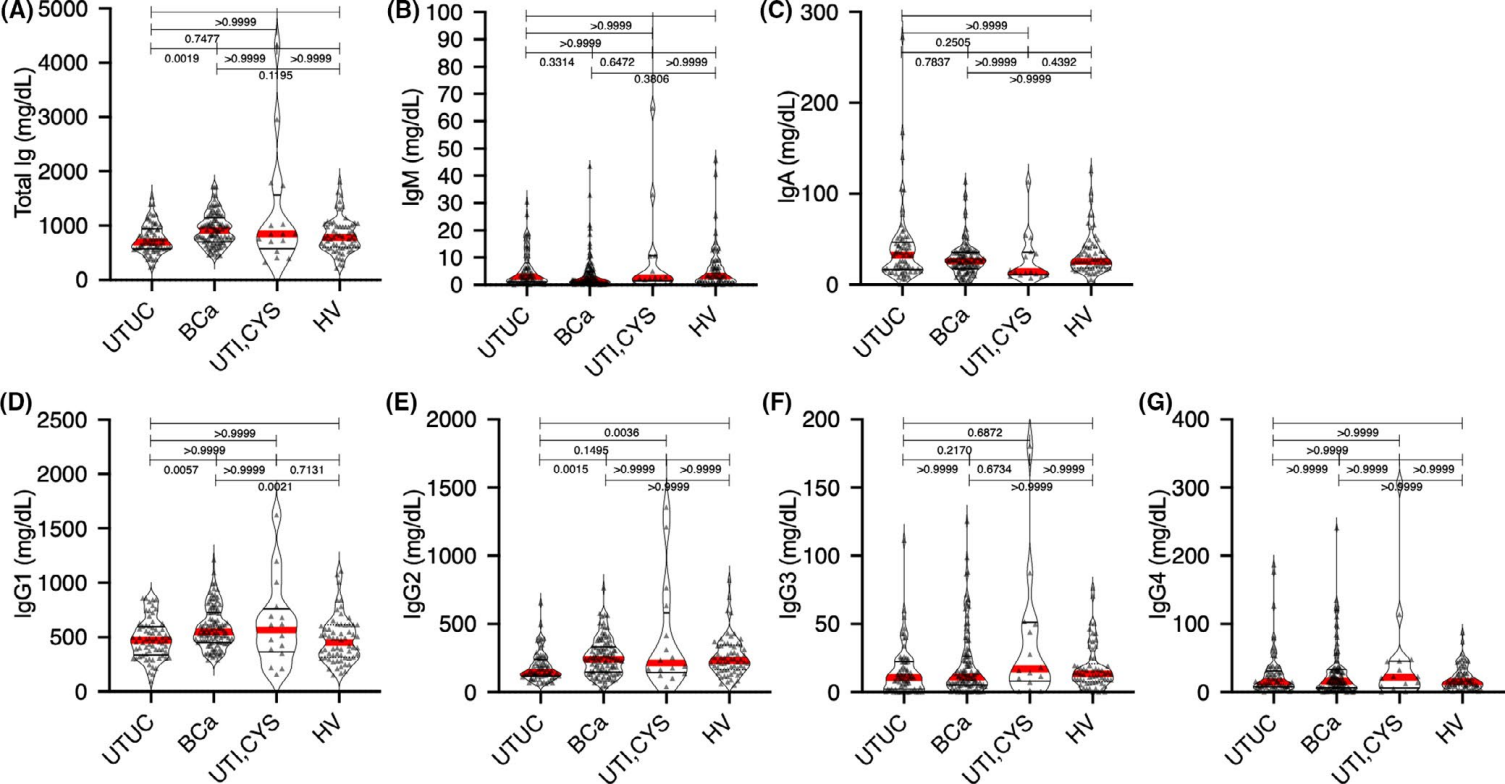
Graphs showing the serum levels of Ig in patients with UTUC, BCa, and UTI, as well as HVs, in the training cohort. (A) Total Ig levels (IgG1–4 + IgM +IgA) in patients with UTUC, BCa, and UTI and HVs. (B) Serum levels of IgM in patients with UTUC, BCa, and UTI, as well as HVs. (C) Serum levels of IgA in patients with UTUC, BCa, and UTI, as well as HVs. (D–G) Serum levels of IgG (IgG1–4) in patients with UTUC, BCa, and UTI, as well as HVs. Data are representative of three independent experiments. Ig, immunoglobulin; UTUC, upper urinary tract urothelial carcinoma; BCa, bladder cancer; UTI, urinary tract infection; HVs, healthy volunteers;

Aliquots (100 μL) of each serum sample were applied to the Zeba™ Spin desalting resin plate (Thermo Fisher Scientific, Waltham, MA, USA) pre‐equilibrated with phosphate‐buffered saline. The plate was then centrifuged at 1000 × *g* for 2 min, after which the flow‐through was collected as a buffer‐exchanged serum (100 μL). Purification of the Ig‐containing fractions was achieved using the Melon™ Gel Spin Purification Kit (Thermo Fisher Scientific) according to the manufacturer's instructions. The buffer‐exchanged serum (100 μL) was applied to the center of the Melon Gel resin pre‐equilibrated with purification buffer. After incubating for 5 min, the Melon Gel resin was centrifuged at 1000 × *g* for 2 min, and the flow‐through was collected as the purified Ig‐containing fraction. A 20‐μL aliquot of this fraction was subjected to *N*‐glycomics analysis. To confirm the purity of the purified Igs, the flow‐through from the Melon Gel resin underwent sodium dodecyl sulfate‐polyacrylamide gel electrophoresis (SDS‐PAGE) analysis and was stained with Coomassie brilliant blue (Figure 2A–D). Total IgG, IgM, and IgA levels in the Ig‐containing fraction were measured using the Bio‐plex Pro Human Isotyping 6‐plex kit (Bio‐Rad Laboratories, Hercules) according to the manufacturer's instructions (Figure 3A–G).

### 2.5 *N*‐glycomics analysis of sera using capillary electrophoresis‐light‐emitting‐diode‐induced fluorescence *N*‐glycan analysis

*N*‐glycomics analysis of sera from patients or HVs was performed using a CE‐LIF‐based Gly‐Q^TM^ *N*‐glycan analysis system (ProZyme, Inc.; Agilent Technologies, Inc.,) combined with the Gly‐X rapid *N*‐glycan preparation method using the automated SweetBlot™ (System Instruments, Hachioji, Japan). Briefly, 20 μL of the Ig‐containing fraction and 2 μL of Gly‐X denaturant were mixed, after which the mixture was denatured by incubating for 3 min at 90°C. After 2 min at room temperature, 2 μL of *N*‐glycanase working solution was added to the denatured samples, followed by incubation for 5 min at 50°C. After this deglycosylation step, 5 μL of InstantQ charged *N*‐glycan dye solution was added to the samples, followed by incubation for 1 min at 50°C. The resulting mixture was loaded onto a prewetted Gly‐X cleanup plate, after which a vacuum of <5 in Hg was applied until the entire sample solution had passed through the membrane. Next, 600 μL of ethanol was loaded onto the Gly‐Q cleanup plate, after which a vacuum of <10 in Hg was applied until all the ethanol had passed through the membrane. This washing step was repeated once. Finally, after adding 150 μL of ultrapure water to each well, the InstantQ‐labeled glycan samples were collected into the collection plate by applying a vacuum of ≤2 in Hg. The InstantQ‐labeled *N*‐glycan was then separated on the Gly‐Q CE system based on LED‐induced fluorescence detection with a run time of 2 min per sample. The Gly‐Q glucose unit (GU) ladder is generally the first run at the beginning of each sample sequence and produces >15 peaks in the electropherogram (Figure S1A). The migration standards from subsequent unknown samples should be aligned to the maltodextrin (GU) calibration ladder for glycan naming and alignment purposes. Migration standards are co‐injected along with each unknown sample. A lower (LMS) and upper migration standard (UMS) corresponding to 3 and 15 glucose units, respectively, was used for alignment with the aforementioned maltodextrin (GU) calibration ladder. The representative electropherogram is presented in Figure S1B–E. The compositions and structures of the glycans were analyzed using the automated peak analysis and glycan assignment from the glycan library functions of the Gly‐Q Manager software (hIgG processing method) (Figure S1).

### 2.6 Statistical analysis

Statistical analyses were performed using BellCurve for Excel®, version 3.20, Stata15/SE (Stata Corp LLC.), GraphPad Prism v.8.41 (GraphPad Software), and R software version 3.5.2 (R Foundation for Statistical Computing; available on: http//www.r‐project.org/). Categorical variables are reported as percentages and were compared using Fisher's exact test, while continuous data are expressed as medians with 25th and 75th quartiles (Q1, Q3). Differences between groups were compared using the Student's *t*‐test for normally distributed data or the Mann–Whitney *U* test for non‐normally distributed data. The Kruskal‐Wallis test was used to analyze differences between multiple groups. Multivariable discriminant analysis was used for the detection of UTUC by inputting UTUC event as an explanatory variable and *N*‐glycan level as an objective variable. The diagnostic *N*‐glycan score was calculated by multiplying candidate *N*‐glycan levels by each discriminant function value. The diagnostic performance of the *N*‐glycan score was evaluated using ROC curve analysis, while statistical differences between the areas under the curves (AUCs) were calculated using the same program.^21^ To assess significant differences between two groups of subjects, a permutation test was conducted to calculate the permutation *p*‐value. Differences with *p* < 0.05 were considered statistically significant. The clinical net benefit of the diagnostic base model, which included gross hematuria status with UTUC or BCa scores for the prediction of UC, was evaluated using DCA.^22^ The “nomlog” command of Stata15/SE software was used to develop the nomogram after building the finalized model using the “logit prediction” command (binary logistic regression model predicting UC). The calibration was estimated using the Hosmer and Lemeshow goodness of fit statistics, while the calibration plot was developed using the Stata command “pmcalplot.” Calibration was assessed by comparing the predicted probability to the observed probability of UC and examined using a calibration plot and calibration slope with 95% confidence intervals (95% CIs). Calibration plots were used to display observed outcomes by deciles of predicted outcome, as well as examine outcomes at the individual level using locally weighted scatterplot smoothing algorithms.^23^ The calibration‐in‐the‐large (CITL) was evaluated to determine whether the predictions were systematically too high or too low.^23^ Given that multiple imputation (MI) data sets were used, the AUC and calibration slope were estimated for each individual MI data set before Rubin's rule was used to combine estimates.^24^

## 3 RESULTS

**FIGURE 4:**
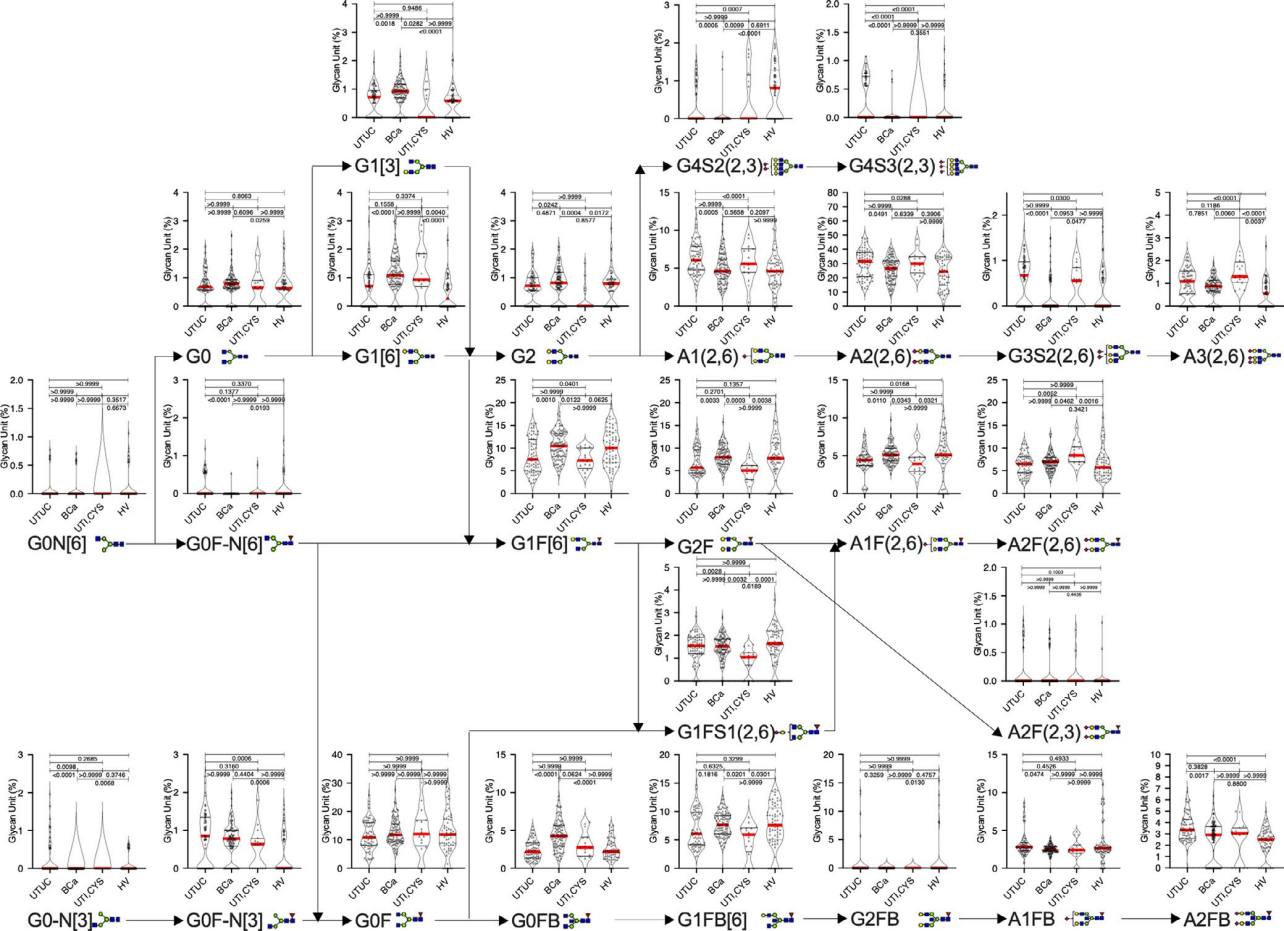
*N*‐glycan signature of immunoglobulins in the training cohort. Serum *N*‐glycans levels in patients with upper urinary tract urothelial carcinoma, bladder cancer, and urinary tract infection, as well as healthy volunteers. BCa, bladder cancer; HV, healthy volunteer; UTI, urinary tract infection; UTUC, upper urinary tract urothelial carcinoma.

A total of 250 subjects were enrolled for analysis: 62 HVs and 68 patients with UTUC, 104 with BCa, 10 with CYS, and 6 with UTI. Patient characteristics in the non‐UC (HV and UTI) and UC (UTUC and BCa) groups are summarized in Table 1. No significant difference in gender and age was noted between the non‐UC and UC groups. SDS‐PAGE analysis of whole serum and Ig‐containing fractions revealed that non‐Ig proteins were effectively eliminated from the whole serum by Melon Gel chromatography (Figure 2A). No significant differences in total Ig, IgA, or IgM levels were observed between both groups (Figure 3). We identified 26 types of *N*‐glycan signatures on Igs using CE‐LIF‐based *N*‐glycomics (Figure 4). However, no UTUC‐ or BCa‐specific aberrant *N*‐glycosylation was found given that individual *N*‐glycan levels did not significantly differ between the groups. To detect UTUC, multivariable discriminant analysis was performed by inputting UTUC as an explanatory variable and whole *N*‐glycan signature (Figure 4) as an objective variable. The UTUC score for the detection of UTUC was established according to results of multivariable discriminant analysis using the following formula (Table 2):

**TABLE 1:** Patient characteristics

Training cohort	Non‐UC group		UC group	
	HV	UTI and Cystitis	UTUC	BCa
Total patients (n)	62	16	68	104
Male sex (n, %)	42 (67.7)	8 (50.0)	45 (66.1)	85 (81.7)
Median age (IQR)	68 (63–74)	80 (69–91)	72 (65–75)	71 (63–76)
Gross hematuria (n, %)	0	2 (12.5)	43 (63.2)	82 (78.8)
Urine Cytology Class (n, %)				
IV, V			33 (48.5)	35 (33.6)
Clinical stage				
Ta			3	3
Tis			1	3
T1			10	61
T2			6	13
T3			32	17
T4			4	8

Validation cohort	Non‐UC group		UC group	
	HV	UTI and Cystitis	UTUC	BCa
Total patients (n)	63	16	68	104
Male sex (n, %)	35 (55.5)	8 (50.0)	48 (70.5)	92 (88.4)
Median age (IQR)	70 (65–75)	75 (69–80)	72 (64–78)	70 (62–76)
Gross hematuria (n, %)	0	2 (12.5)	40 (58.8)	78 (75.0)
Urine Cytology Class (n, %)				
IV, V			26 (38.2)	43 (41.3)
Clinical stage				
Ta			2	2
Tis			0	3
T1			14	59
T2			12	18
T3			28	18
T4			1	3

**TABLE 2:** Multivariable discriminant analysis for the prediction of UTUC and BCa

UTUC vs HV, UTI and Cystitis						
Variables	Wilks' lambda	F value	ODF	TDF	*p* value	Discriminant function
A3(2,6)	0.970	4.423	1	144	0.037	0.368
A2(2,6)*	0.959	6.160	1	144	0.014	0.041
A2F(2,6)	0.986	2.051	1	144	0.154	0.085
A2FB(2,6)*	0.827	30.060	1	144	<0.001	−0.4745
G4S3(2,3)*	0.859	23.686	1	144	<0.001	−1.111
G3S2(2,6)	0.958	6.265	1	144	0.013	0.326
G1FS1(2,6)	1.000	0.023	1	144	0.879	−0.184
A1(2,6)*	0.893	17.331	1	144	<0.001	−0.142
G0‐N[6]	0.998	0.293	1	144	0.589	0.554
A1F(2,6)	0.985	2.213	1	144	0.139	0.228
A1FB(2,6)	0.998	0.293	1	144	0.589	0.343
G0F‐N[6]	0.997	0.409	1	144	0.523	0.284
G0F‐N[3]*	0.912	13.965	1	144	<0.001	0.265
G0	0.980	2.867	1	144	0.093	−0.712
G0F	0.996	0.567	1	144	0.453	0.161
G0FB	0.997	0.424	1	144	0.516	0.089
G1[6]	0.999	0.197	1	144	0.658	−0.106
G1[3]	1.000	0.000	1	144	0.996	0.531
G1F[6]	0.975	3.654	1	144	0.058	0.022
G1FB[6]	0.999	0.105	1	144	0.747	−0.205
G2	0.994	0.814	1	144	0.368	0.266
G2F	0.997	0.424	1	144	0.516	0.127
G2FB	0.999	0.185	1	144	0.668	0.088
G0‐N[3]*	0.943	8.745	1	144	0.004	−0.624
G4S2(2,3)*	0.929	11.071	1	144	0.001	0.699
A2F(2,3)*	0.964	5.336	1	144	0.022	−0.709
Constant term						−3.987

BCa vs HV, UTI and Cystitis						
Variables	Wilks' lambda	F value	ODF	TDF	*p* value	Discriminant function
A3(2,6)	0.994	1.077	1	180	0.301	−0.255
A2(2,6)	1.000	0.069	1	180	0.793	0.039
A2F(2,6)	0.995	0.817	1	180	0.367	0.011
A2FB(2,6)	1.000	0.015	1	180	0.904	−0.260
G4S3(2,3)*	0.970	5.641	1	180	0.019	0.355
G3S2(2,6)*	0.936	12.215	1	180	0.001	−0.997
G1FS1(2,6)	1.000	0.075	1	180	0.784	0.655
A1(2,6)	0.998	0.327	1	180	0.568	0.152
G0‐N[6]	0.992	1.362	1	180	0.245	0.158
A1F(2,6)	1.000	0.081	1	180	0.777	0.003
A1FB(2,6)*	0.976	4.362	1	180	0.038	−0.014
G0F‐N[6]*	0.953	8.796	1	180	0.003	−0.020
G0F‐N[3]*	0.887	22.983	1	180	<0.001	0.754
G0	0.968	5.974	1	180	0.015	0.127
G0F	1.000	0.012	1	180	0.914	−0.159
G0FB*	0.796	46.049	1	180	<0.001	0.595
G1[6]*	0.869	27.204	1	180	<0.001	0.136
G1[3]	0.968	6.035	1	180	0.015	−0.119
G1F[6]	0.984	2.964	1	180	0.087	0.051
G1FB[6]	0.989	1.949	1	180	0.164	0.028
G2*	0.970	5.560	1	180	0.019	−0.062
G2F	0.975	4.634	1	180	0.033	−0.092
G2FB	0.958	7.974	1	180	0.005	−0.307
G0‐N[3]*	0.915	16.767	1	180	<0.001	−2.319
G4S2(2,3)*	0.731	66.367	1	180	<0.001	−1.018
A2F(2,3)	0.986	2.576	1	180	0.110	1.228
Constant term						−2.006

UTUC score = (serum level of A3[2,6] × 0.368) + (serum level of A2[2,6] × 0.041) + (serum level of A2F[2,6] × 0.085) + (serum level of A2FB[2,6] × −0.474) + (serum level of G4S3[2,3] × −1.112) + (serum level of G3S2[2,6] × 0.326) + (serum level of G1FS1[2,6] × −0.184) + (serum level of A1[2,6] × −0.142) + (serum level of G0‐N[6] × 0.554) + (serum level of A1F[2,6] × 0.228) + (serum level of A1FB[2,6] × 0.343) + (serum level of G0F‐N[6] × 0.284) + (serum level of G0F‐N[3] × 0.265) + (serum level of G0 × −0.712) + (serum level of G0F × 0.161) + (serum level of G0FB × 0.089) + (serum level of G1[6] × −0.107) + (serum level of G1[3] × 0.531) + (serum level of G1F[6] × 0.022) + (serum level of G1FB[6] or G1F[3] × −0.206) + (serum level of G2 × 0.266) + (serum level of G2F × 0.127) + (serum level of G2FB × 0.088) + (serum level of G0‐N[3] × −0.624) + (serum level of G4S2[2,3] × 0.699) + (serum level of A2F[2,3] × −0.709) + (−3.987).

To detect BCa, multivariable discriminant analysis was performed by inputting BCa as an explanatory variable and whole *N*‐glycan signature as an objective variable. The BCa score was calculated according to the results of multivariable discriminant analysis using the following formula (Table 2):

BCa score = (serum level of A3[2,6] × −0.256) + (serum level of A2[2,6] × 0.039) + (serum level of A2F[2,6] × 0.011) + (serum level of A2FB[2,6] × −0.260) + (serum level of G4S3[2,3] × 0.355) + (serum level of G3S2[2,6] × −0.998) + (serum level of G1FS1[2,6] × 0.655) + (serum level of A1[2,6] × 0.152) + (serum level of G0‐N[6] × 0.158) + (serum level of A1F[2,6] × 0.003) + (serum level of A1FB[2,6] × −0.014) + (serum level of G0F‐N[6] × −0.021) + (serum level of G0F‐N[3] × 0.754) + (serum level of G0 × 0.127) + (serum level of G0F × −0.160) + (serum level of G0FB ×0.595) + (serum level of G1[6] × 0.136) + (serum level of G1[3] × −0.120) + (serum level of G1F[6] × 0.051) + (serum level of G1FB[6] or G1F[3] × 0.028) + (serum level of G2 × −0.062) + (serum level of G2F × −0.092) + (serum level of G2FB × −0.307) + (serum level of G0‐N[3] × −2.319) + (serum level of G4S2[2,3] × −1.018) + (serum level of A2F[2,3] × 1.228) + (−2.006).

**FIGURE 5:**
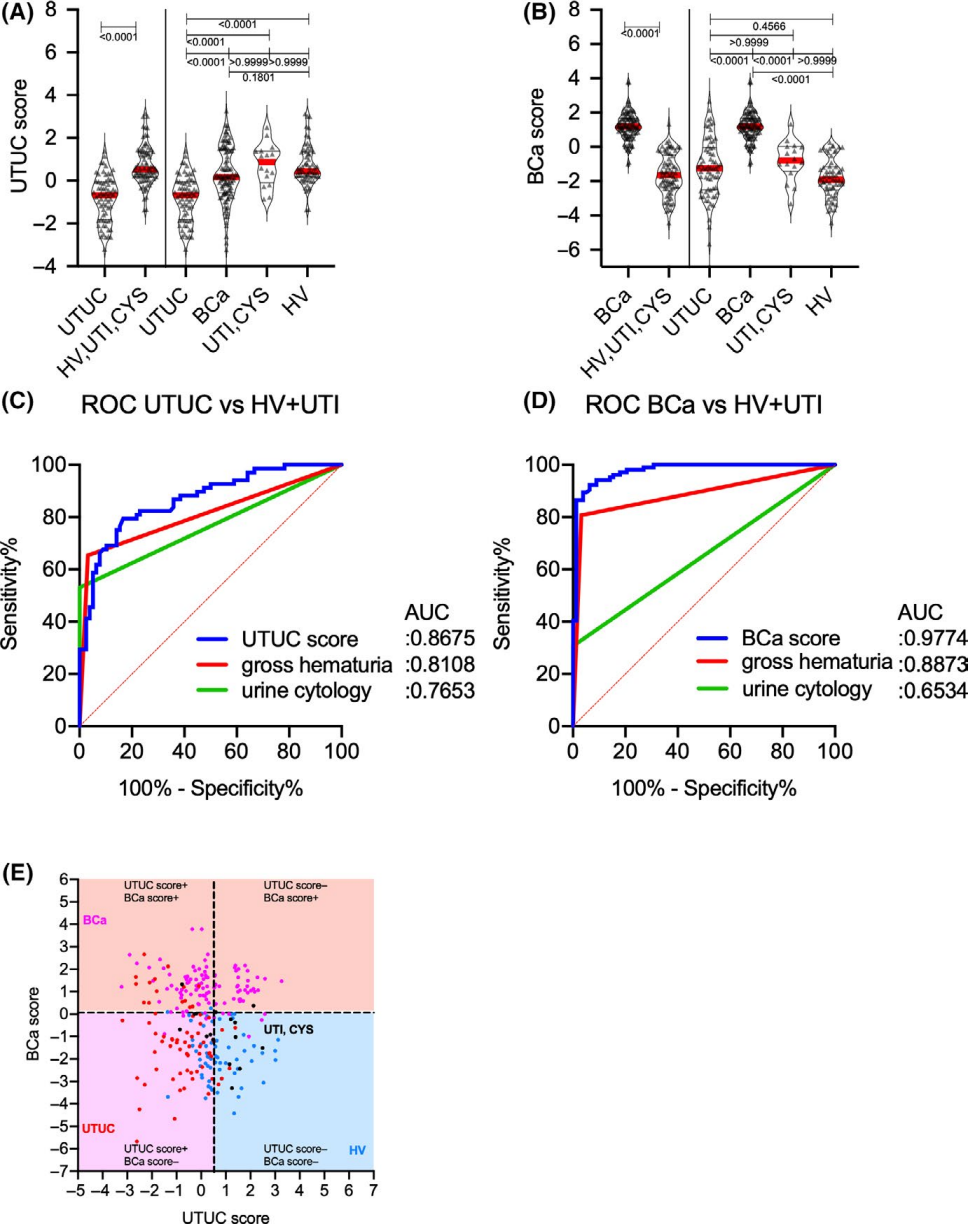
Upper urinary tract urothelial carcinoma and bladder cancer scores for the detection of the conditions in the training cohort. (A) UTUC score levels in patients with UTUC, BCa, and UTI, as well as HVs. (B) BCa score levels in patients with UTUC, BCa, and UTI, as well as HVs. (C) ROC curve analysis of UTUC score, gross hematuria, and urine cytology results for the detection of UTUC. (D) ROC curve analysis of BCa score, gross hematuria, and urine cytology for the detection of BCa. (E) Scatter diagram of UTUC score and BCa score. AUC, area under curve; BCa, bladder cancer; HV, healthy volunteer; UTI, urinary tract infection; UTUC, upper urinary tract urothelial carcinoma; ROC curve, receiver operating characteristic curve.

**FIGURE 6:**
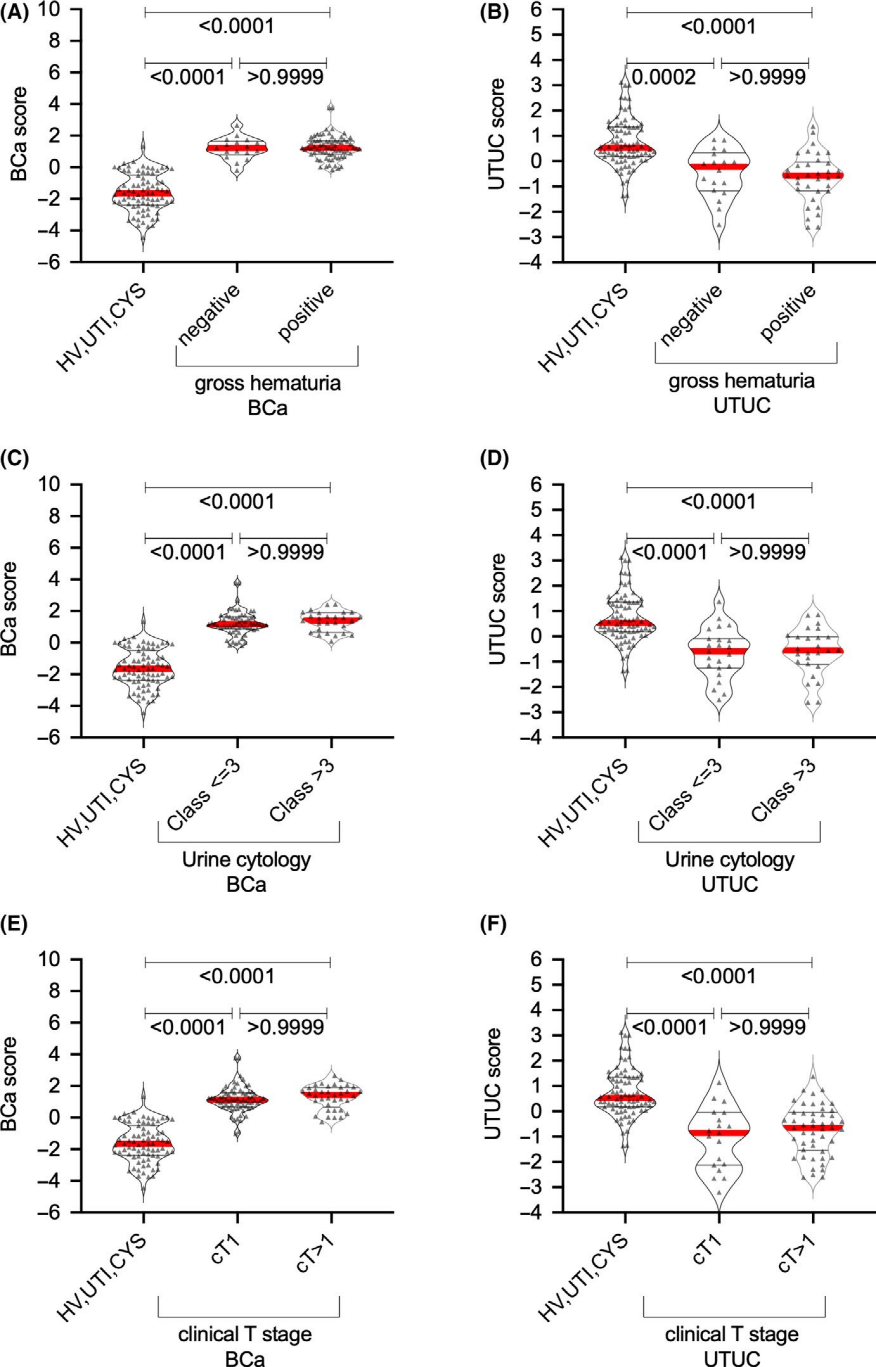
Association between upper urinary tract urothelial carcinoma and bladder cancer scores and gross hematuria status, urine cytology, and clinical T stage in the training cohort. Association between (A) UTUC score or (B) BCa score and gross hematuria status. Association between (C) UTUC score or (D) BCa score (D) and urine cytology status. Association between (E) UTUC score or (F) BCa score and clinical T stage. BCa, bladder cancer; HV, healthy volunteer; UTI, urinary tract infection; UTUC, upper urinary tract urothelial carcinoma.

Each cancer group had significantly different median UTUC and BCa scores than the non‐UC group (Mann–Whitney *U* test: *p* < 0.001) (Figure 5A,B). AUCs of the UTUC and BCa scores for predicting of UTUC and BCa were 0.868 (95% Cl 0.809–0.926) and 0.977 (95% Cl 0.958–0.996), respectively, which were significantly higher than that of urine cytology (UTUC: 0.765, 95% Cl 0.669–0.861; BCa: 0.653, 95% Cl 0.568–0.739) or gross hematuria (UTUC: 0.811, 95% Cl 0.728–0.897; BCa: 0.887, 95% Cl 0.831–0.944) (Figure 5C,D). Both UTUC and BCa scores could be calculated in patients with UC despite negative urine cytology and/or hematuria and/or clinical T1 status (Figure 6A–F, Table 3). At the preset specificity of 90%, the UTUC score had a sensitivity, positive predictive value (PPV), and negative predictive value (NPV) of 67.6%, 86.8%, and 76.3% for detecting UTUC, while the BCa score had a sensitivity, PPV, and NPV of 94.2%, 93.3%, and 92.2% for detecting BCa, respectively (Table 4).

**TABLE 3:** Receiver operating characteristic curve analyses of urine cytology, hematuria, and all scores

		*p*‐value	
UTUC detection	AUC (95% Cl)	vs UTUC score	vs UTUC score
Urine cytology	0.765 (0.669–0.861)	0.002	
Gross hematuria	0.810 (0.724–0.896)		0.125
UTUC score	0.867 (0.809–0.925)		

BCa detection	AUC (95%Cl)	vs BCa score	vs BCa score
Urine cytology	0.653 (0.567–0.739)	<0.001	
Gross hematuria	0.887 (0.831–0.943)		<0.001
BCa score	0.977 (0.958–0.996)		

**TABLE 4:** Specificity, positive predictive value, and negtive predictive value at 90% sensitivity of each assay in the training cohort

	UTUC score for UTUC detection	BCa score for BCa detection
Cut‐off	<0.4550	>0.0675
AUC (95%Cl)	0.868 (0.809–0.926)	0.977 (0.958–0.996)
Specificity, % (95%Cl)	67.6 (55.8–77.5)	94.2 (85.9–97.2)
PPV, %	86.8	93.3
NPV, %	76.3	92.2

PPV, positive predictive value; NPV, negative predictive value

**FIGURE 7:**
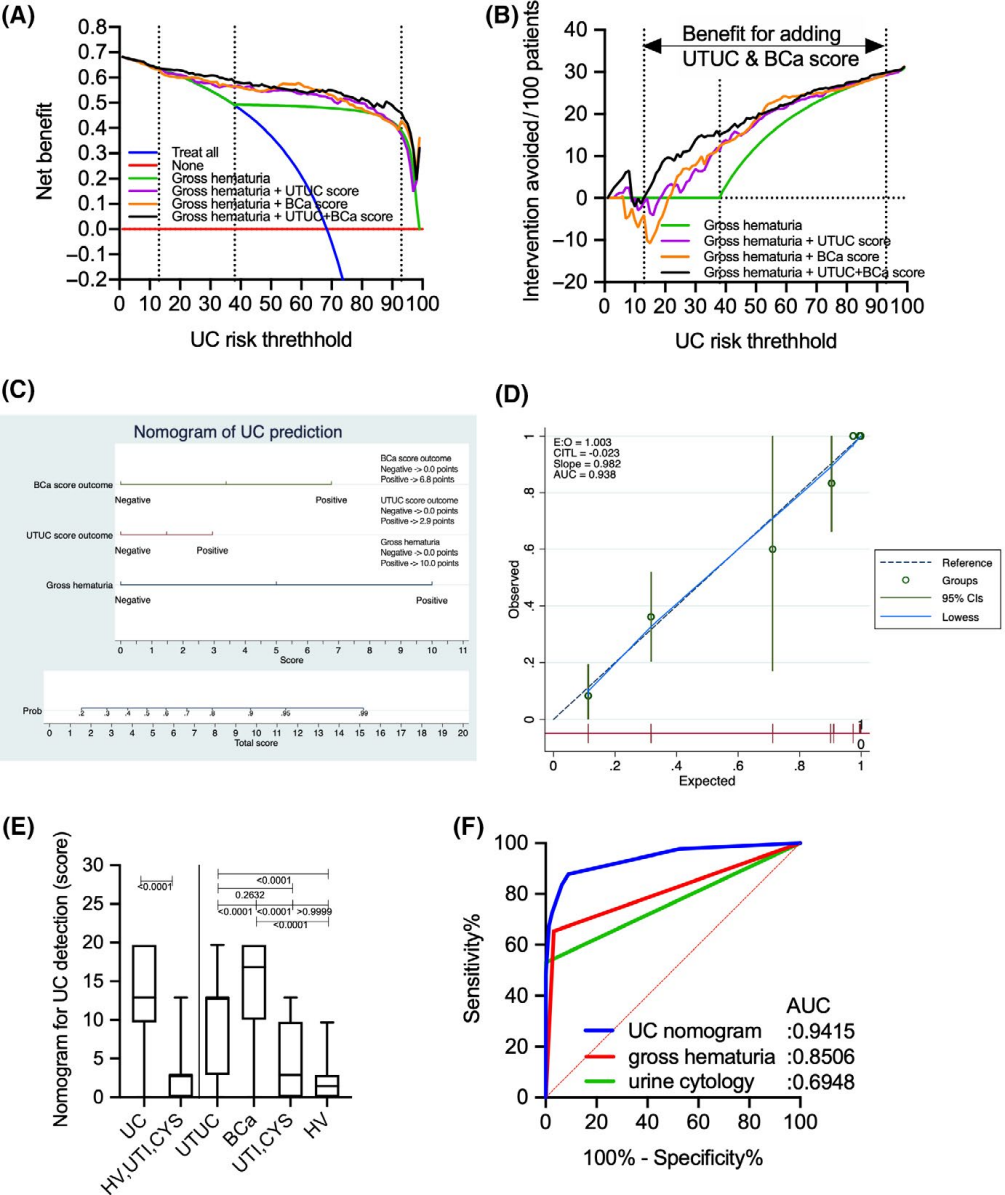
Comparison of decision curve plots with net benefit for the relevant risk threshold between the base model (gross hematuria), base model +upper urinary tract urothelial carcinoma score, base model +bladder cancer score, and base model +upper urinary tract urothelial carcinoma +bladder cancer scores in the training cohort. (A) Decision curve plots showing the net benefit for detecting urothelial carcinoma (UC). (B) Decision curve plots showing the intervention avoided per 100 patients; plots were developed using the rmda package of R statistical software. (C) Logistic regression analysis‐based nomogram for the prediction of UC. (D) Calibration plot depicting the calibration of the UC nomogram. (E) Boxplot of UC and non‐UC (HV, UTI, and CYS) based on the UC nomogram, gross hematuria, and urine cytology. (F) Receiver operating characteristic curve analysis of the UC nomogram, gross hematuria, and urine cytology for the prediction of UC. UC, urothelial carcinoma; BCa, bladder cancer; HV, healthy volunteer; UTI, urinary tract infection.

DCA for predicting overall UC revealed that gross hematuria status combined with UTUC and BCa scores had the largest net benefit for overall UC prediction, with a 13%–93% risk threshold (Figure 7A). At a risk threshold of 38%, the avoidance of intervention without overlooking UC promoted by gross hematuria status combined with all discriminant scores (15/100 patients) significantly improved the gross hematuria status (0/100 patients) (Figure 7B, Table 5).

**TABLE 5:** Net benefit and avoidable intervention for the diagnostic model compared to the treat‐all strategy

	Diagnostic model				Diagnostic model			
	Base model (Gross hematuria)				Base model (Gross hematuria)			
		+UTUC score	+BCa score	+UTUC+BCa score		+UTUC score	+BCa score	+UTUC+BCa score
UC risk threshhold (%)	Net benefit for detecting UC				Interventions avoided/100 patients			
5	0.67	0.67	0.67	0.67	0	1	0	4
10	0.65	0.65	0.64	0.65	0	0	0	0
15	0.63	0.62	0.61	0.63	0	0	0	3
20	0.61	0.61	0.60	0.63	0	2	0	9
25	0.58	0.58	0.60	0.61	0	1	6	10
30	0.55	0.58	0.58	0.61	0	7	8	14
35	0.51	0.57	0.57	0.59	0	10	11	15
40	0.49	0.57	0.56	0.58	3	14	13	16
45	0.49	0.55	0.55	0.57	8	15	15	18
50	0.49	0.55	0.55	0.56	12	18	19	19
55	0.49	0.55	0.57	0.55	16	21	23	21
60	0.49	0.53	0.57	0.54	18	22	24	22
65	0.48	0.53	0.55	0.55	21	23	24	24
70	0.48	0.52	0.53	0.55	23	24	25	26
75	0.47	0.51	0.51	0.53	25	26	26	26
80	0.47	0.50	0.48	0.52	26	27	26	27
85	0.45	0.46	0.45	0.50	27	28	27	28
90	0.43	0.42	0.42	0.49	29	29	29	29
95	0.34	0.31	0.39	0.42	30	30	30	30
99	0.00	0.32	0.36	0.32	31	31	31	31

**FIGURE 8:**
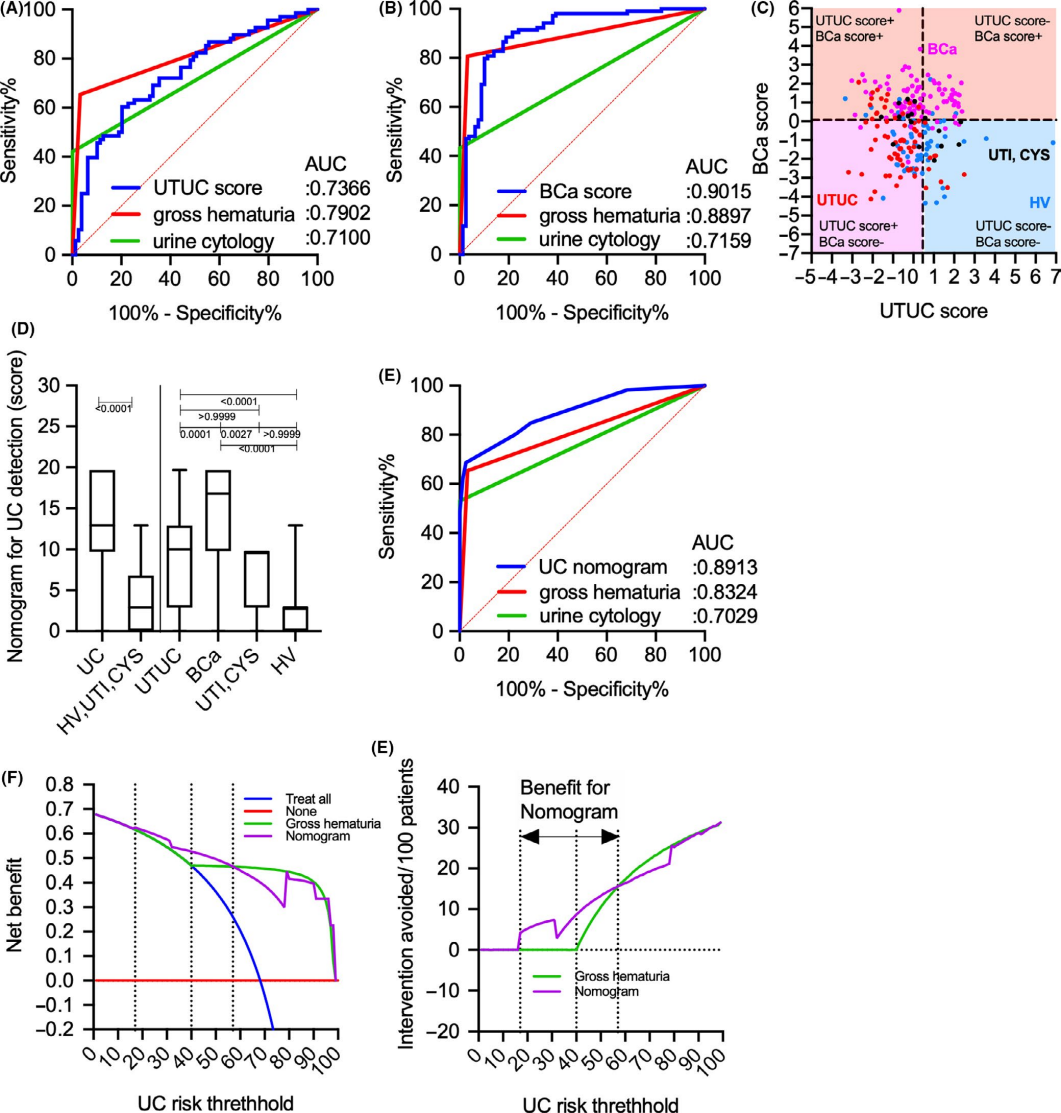
UTUC or BCa scores for the detection of conditions and comparison of decision curve plots with net benefit for the relevant risk threshold between the base model (gross hematuria) and the UC nomogram in the validation cohort. (A) ROC curve analysis of the UTUC score, gross hematuria, and urine cytology for the detection of UTUC. (B) ROC curve analysis of the BCa score, gross hematuria, and urine cytology for the detection of BCa. (C) Scatter diagram of the UTUC and BCa scores. (D) Boxplot of UC and non‐UC (HV, UTI, and CYS) based on the UC nomogram, gross hematuria, and urine cytology. (E) ROC curve analysis of the UC nomogram, gross hematuria, and urine cytology for the prediction of UC. (F) Decision curve plots showing the net benefit for detecting UC. (G) Decision curve plots showing the intervention avoided per 100 patients; plots were developed using the rmda package of R statistical software. AUC, area under curve; BCa, bladder cancer; CI, confidence interval; CITL, calibration‐in‐the‐large; HV, healthy volunteer; PPV, positive predictive value; NPV, negative predictive value; ROC, receiver operating characteristic; UC, urothelial carcinoma. UTI, urinary tract infection; UTUC, upper urinary tract urothelial carcinoma.

Based on DCA results, gross hematuria status, UTUC score, and BCa score were selected for the prediction of UC outcomes. Table 6 shows the results of multivariate logistic regression analysis for UC prediction. The model with the selection criteria of *p* > 0.5 was selected as the best model due to better goodness of fit statistics (pseudo R^2^: 0.566, Akaike information criterion: 142.85, Bayesian information criterion: 156.94, ROC area: 0.925, Hosmer and Lemeshow *p* = 0.096). Gross hematuria status [odds ratio (OR): 80.33, *p* < 0.001], UTUC score (OR: 3.65, *p* = 0.007), and BCa score (OR: 19.48; *p* < 0.001) were selected for the model, and a nomogram was developed based on the results of multivariate analysis (Figure 7C, Table 6). The calibration plots presented in Figure 7D show that the nomogram‐predicted probabilities of UC were similar to the actual probabilities of UC, indicating that the prediction had good agreement with actual incidence of UC (calibration slope =0.982). The CITL, which should preferably be zero, indicated that the difference between the observed prevalence and the mean UC nomogram‐predicted probability was too high (CITL = −0.023), suggesting that the discrimination ability of the nomogram for predicting UC could be generalizable to other populations and may be clinically applicable. UC patients had significantly higher scores based on UC nomogram than non‐UC (HV, UTI, and CYS) patients (*p* < 0.001) (Figure 7E). The AUC of the UC nomogram for predicting UC (0.941, 95% Cl 0.914–0.969) was significantly higher than that of urine cytology (0.695, 95% Cl 0.631–0.759) and gross hematuria (0.851, 95% Cl 0.803–0.898) (Figure 7F). The sensitivity, specificity, PPV, NPV, false‐positive rate, and false‐negative rate of the UC nomogram for detecting UC were 87.8%, 91.1%, 95.6%, 77.4%, 8.9%, and 12.2%, respectively. In the validation cohort, the AUCs of the UTUC and BCa scores for predicting UTUC and BCa were 0.736 (95% Cl 0.655–0.817) and 0.901 (95% Cl 0.853–0.949) (Figure 8A, B). Both UTUC and BCa scores could detect patients with UC despite negative urine cytology and/or hematuria and/or clinical T1 status (Figure S2). The combination of UTUC and BCa scores was able to clearly discriminate UTUC and BCa from UTI, CYS, or healthy status (Figure 8C). Patients with UC had significantly higher boxplot results based on the UC nomogram than non‐UC (HV, UTI, and CYS) patients (*p* < 0.001) (Figure 8D). The AUC of the UC nomogram for predicting UC (0.891, 95% Cl 0.853–0.929) was significantly higher than that of urine cytology (0.710, 95% Cl 0.608–0.811) and gross hematuria (0.790, 95% Cl 0.701–0.879) (Figure 8E). DCA for predicting overall UC showed that the nomogram had the largest net benefit for overall UC prediction, with a 15%–57% risk threshold (Figure 8F). At a risk threshold of 40%, the avoidance of intervention without overlooking UC promoted by gross hematuria status combined with all discriminant scores (9/100 patients) significantly improved the gross hematuria status (0/100 patients) (Figure 8G, Table 7).

**TABLE 6:** Multivariate logistic regression model for UC prediction

UC outcome	Odds Ratio	Std. Err.	z	*p*‐value	[95% CI]	
Gross hematuria	80.33	61.82	5.7	0.000	17.77	363.03
UTUC score	3.64	1.73	2.72	0.007	1.43	9.27
BCa score	19.48	10.99	5.26	0.000	6.44	58.88
_cons	0.12	0.05	–4.79	0.000	0.05	0.29
Log likelihood = 175.49			Pseudo R2 = 0.5655			

UC detection	AUC (95% Cl)	P value vs UC nomogram
Urine cytology^a^	0.694 (0.630–0.758)	<0.001
Gross hematuria^b^	0.850 (0.802–0.898)	<0.001
UC nomogram^c^	0.941 (0.914–0.969)	

**TABLE 7:** Net benefit and avoidable intervention for the diagnostic model compared to the treat‐all strategy

	Diagnostic model		Diagnostic model	
	Gross hematuria	UC Nomogram	Gross hematuria	UC Nomogram
UC risk threshhold (%)	Net benefit for detecting UC		Intervention avoided/100 patients	
5	0.66	0.66	0	0
10	0.65	0.65	0	0
15	0.63	0.63	0	0
20	0.60	0.61	0	5
25	0.58	0.60	0	6
30	0.54	0.58	0	7
35	0.51	0.54	0	5
40	0.47	0.53	0	9
45	0.47	0.51	6	11
50	0.47	0.50	11	13
55	0.47	0.48	14	15
60	0.46	0.45	17	16
65	0.46	0.42	20	18
70	0.46	0.39	22	19
75	0.45	0.34	24	20
80	0.44	0.42	26	25
85	0.43	0.41	27	27
90	0.40	0.40	29	29
95	0.32	0.33	30	30
99	0.00	0.00	31	31

## 4 DISCUSSION

Several studies involving high‐throughput, comprehensive, and quantitative *N*‐glycomics analyses have shown that differences in serum *N*‐glycan profiles between benign and malignant conditions were useful for the diagnosis or prognostication of diseases.^8^, ^9^, ^10^, ^11^, ^15^ Some studies have investigated the use of certain serum *N*‐glycans as diagnostic markers for UC, as well as UTUC.^10^, ^15^ Although the aforementioned reports have demonstrated an increase in levels of highly branched sialylated *N*‐glycans (*m*/*z* 2890, 3560, and 3865) in the sera of patients with bladder cancer,^10^, ^15^ they did not identify specific proteins with aberrant *N*‐glycosylation. Furthermore, these highly branched sialylated *N*‐glycans were found to be significantly upregulated in some cancers, such as prostate or kidney cancers.^8^, ^9^ We had previously focused on *N*‐glycomics analysis of major *N*‐glycosylated proteins in sera, such as Igs. Accordingly, our findings revealed that a total of five types of *N*‐glycans, including bisecting‐GlcNAc‐biantennary‐type *N*‐glycans, with or without core fucose in Ig‐containing fractions of the sera were associated with UC detection.^16^ Moreover, we found that asialo bisecting GlcNAc type *N*‐glycans on Igs are significantly accumulated in patients with UC. However, the current study investigated only five types of *N*‐glycan signatures on Igs, including biantennary and bisecting *N*‐glycans. Although no UTUC‐ or BCa‐specific aberrant *N*‐glycosylation had been detected herein, our results showed that major *N*‐glycan modifications, such as sialylation, fucosylation, bisecting GlcNAcylation, and multi‐branching, were regulated by the balance of various glycosyltransferase activities, which might influence each other by inhibiting associated reactions. Thus, we speculated that the overall *N*‐glycosylation signature could differ between benign and malignant conditions, much like a fingerprint. Glycosylation of Igs has been known to play a critical role in disease development. Accordingly, Wuhrer et al. reported that asialo bisecting type *N*‐glycosylated IgG induces an anti‐inflammatory response, whereas agalactosyl‐bisecting‐type *N*‐glycosylated IgG induces a pro‐inflammatory response in cerebrospinal fluid.^25^ Furthermore, studies have shown that the overproduction of aberrantly glycosylated immunoglobulin A1 plays a key role in the development of IgA nephropathy.^26^ Antibody‐mediated rejection following kidney transplantation has been closely associated with the levels of immunomodulatory sialylated IgG antibodies,^27^ while α2,6‐sialylated IgG levels have been shown to decrease significantly in the context of prostate cancer immunoreactions.^28^ Rademacher et al. reported that agalactosyl‐bisecting‐GlcNAc‐type *N*‐glycosylated IgG levels increased in a patient with rheumatoid arthritis and related autoimmune disease.^29^ Ferdosi et al. reported that patients with BCa, including former ones, had significantly higher levels of α2,6 sialylation, β1,4 branching, β1,6 branching, and outer‐arm fucosylation in plasma Igs compared to healthy living kidney donors.^30^

Aberrantly glycosylated Igs appear to change their glycans due to disease‐associated immunoreactions. Thus, analyzing the overall *N*‐glycosylation signatures in benign and cancerous conditions is important. The present study identified 26 types of *N*‐glycan levels, including biantennary, triantennary, tetraantennary, fucosylated biantennary, and fucosylated‐bisecting‐GlcNAc *N*‐glycans, using high‐throughput rapid CE‐LIF‐based *N*‐glycan analysis. We also established UTUC and BCa scores based on disease‐associated *N*‐glycan signatures on Igs (Figure 5). The combination of UTUC and BCa scores were able to clearly discriminate UTUC and BCa from UTI, CYS or healthy status in the training and validation cohorts (Figure 5E, Figure 8C). Discriminant analysis showed that significantly weighted UTUC‐specific *N*‐glycans (di‐ or mono‐sialylated biantennary glycans A2[2,6], A2FB[2,6], A1[2,6], A2F[2,3]) differed from those of BCa (mono‐sialylated bisecting glycan A1FB[2,6]; galactosylated bisecting glycan G0FB; and galactosylated biantennary glycans G1[6] and G2) (Table 2). This suggests that the *N*‐glycan signature of UTUC differed from that of BCa, which may reflect differences in the tumor environment between the bladder and urinary/renal pelvis and/or the embryonic background of the tumor. Our results also indicated that the UTUC and BCa scores were not related to urine cytological classification, gross hematuria status, and clinical T stage in the training and validation cohorts (Figure 6, Figure S2), suggesting a higher predictive value for cases that are urine cytology negative (<Class IV) or gross hematuria negative. Moreover, the aforementioned scores can be used to detect small tumors otherwise difficult to detect using imaging modalities, which might allow for the early detection of UC prior to urine cytology or imaging examinations. In addition, DCA showed that the combination of gross hematuria status, UTUC score, and BCa score significantly reduced the need for intervention, with its clinical impact being significantly better than preliminary screening for gross hematuria (Figure 7A, B). The established UC nomogram based on gross hematuria status, UTUC score outcome at 90% specificity, and BCa score outcome at 90% specificity also showed goodness of fit with the calibration plot (Figure 7C, D). ROC analysis of the UC nomogram revealed significantly better discrimination compared to urine cytology and gross hematuria status in the training cohort data set. In the validation cohort, DCA showed that the use of the UC nomogram significantly reduced the need for interventions, with its clinical impact being significantly better than preliminary screening for gross hematuria status (Figure 8F, G). The aforementioned results suggest that the UC nomogram, UTUC score, and BCa score might be clinically useful for the early diagnosis of UC and for discriminating between UTUC and BCa.

Some limitations of the present study need to be acknowledged. First, this study included a small sample size and was retrospective in nature, which could lead to selection bias. Furthermore, the study lacked an independent validation group. Therefore, the results obtained herein should be considered preliminary, and further external validation studies are needed. Considering the unreliability of urine cytology for detecting early stage UCs, including UTUC, large‐scale prospective validation studies involving natural cohorts of patients with hematuria are warranted. Moreover, this study excluded patients with benign diseases, such as calculi and prostatitis. Despite these limitations, the strength of the present study lies in our use of a diagnostic nomogram for UC, including UTUC and BCa, based on gross hematuria status, which enabled high‐throughput rapid CE‐LIF‐based *N*‐glycan analysis using a one‐time serum collection. Our previous MS‐based *N*‐glycomics approach required 48 to 72 h to identify the *N*‐glycan structure of Igs and lacked sufficient versatility for clinical application. Furthermore, MS‐based *N*‐glycomics requires substantial costs. The use of CE‐LIF‐based *N*‐glycan analysis overcomes the aforementioned issues. Overall, the findings presented herein may enable the detection of UC even among patients who have negative urine cytology for UC. Nonetheless, further external validation trials are needed to validate the application of this technique in routine clinical practice.

## CONFLICTS OF INTEREST

The authors have no conflicts of interest.

## Supporting information

Fig S1

Fig S2

## Data Availability

The data that support the findings of this study are available from the corresponding author, Tohru Yoneyama, upon reasonable request.
