# Supplementary figures and images for: N‐glycan signature of serum immunoglobulins as a diagnostic biomarker of urothelial carcinomas
Source: Cancer Med. 2021 Jan 16;10(4):1297–313. doi: 10.1002/cam4.3727 (PMC7926015; doi:10.1002/cam4.3727)

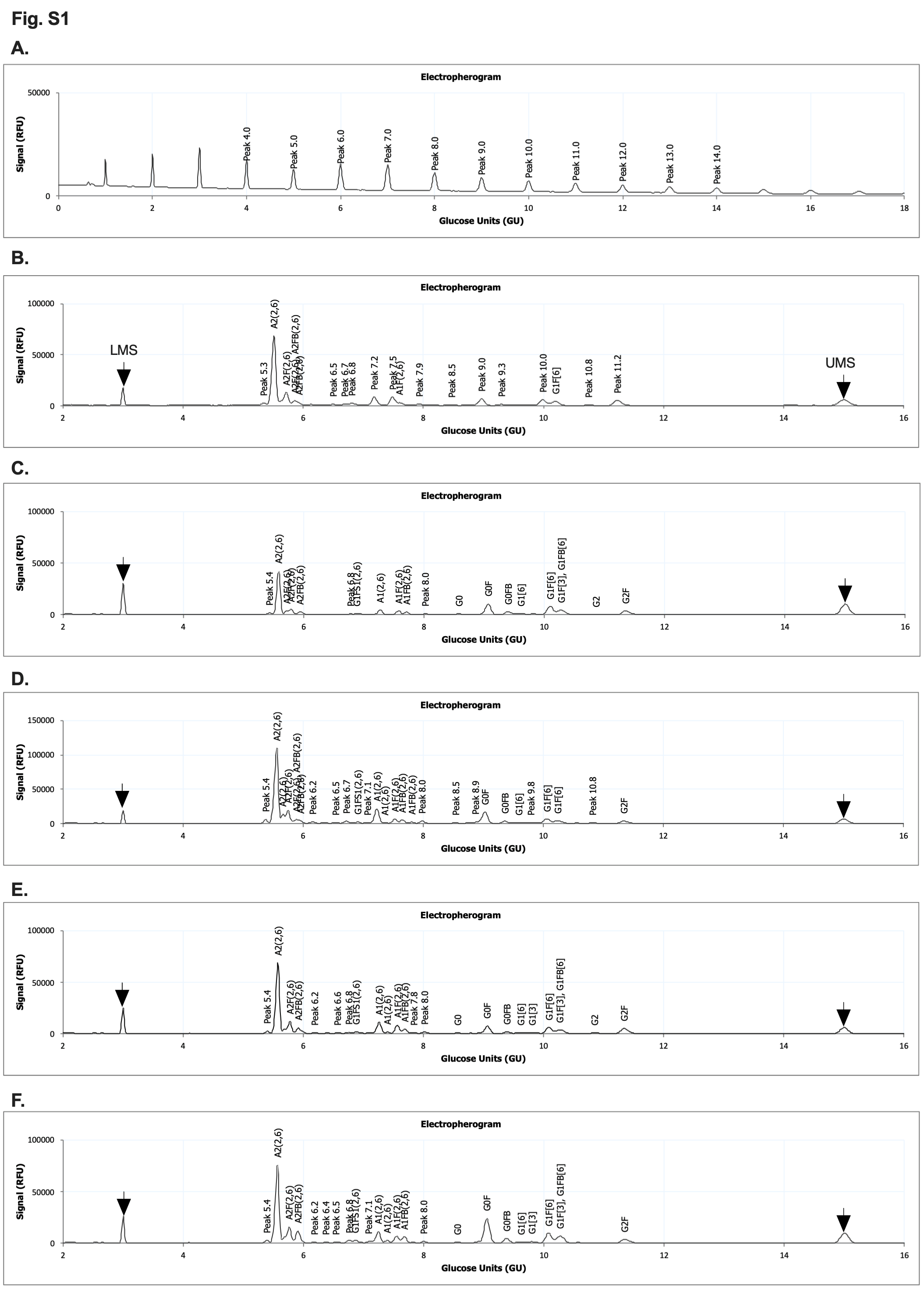

Supplement: Supplementary file 1 — Fig S1 [file CAM4-10-1297-s001.tiff]

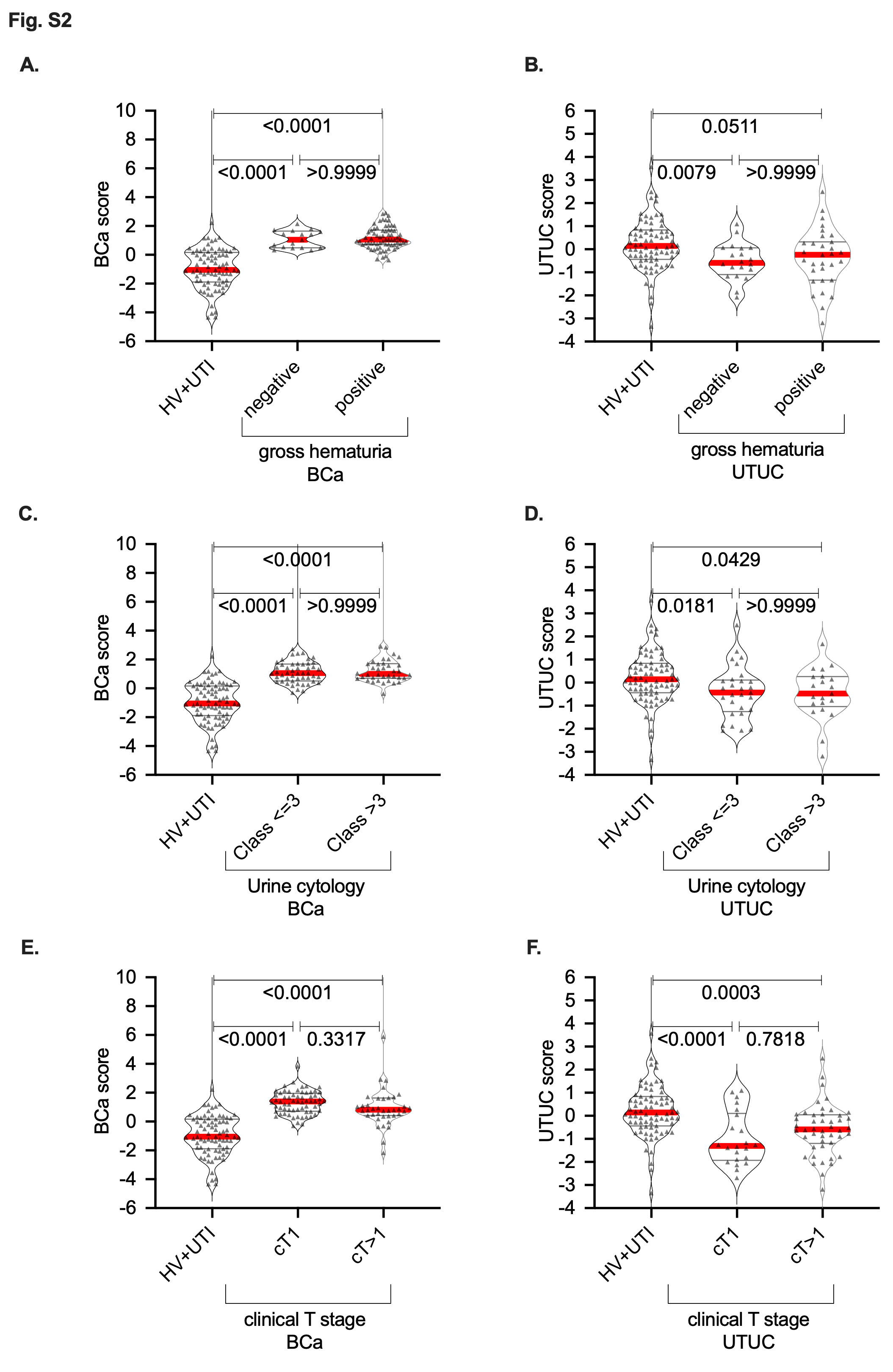

Supplement: Supplementary file 2 — Fig S2 [file CAM4-10-1297-s002.tiff]
